# The influence of pupil responses on subjective brightness perception

**DOI:** 10.1177/03010066221094757

**Published:** 2022-05-02

**Authors:** I. K. Wardhani, C. N. Boehler, S. Mathôt

**Affiliations:** 26656 Ghent University, Belgium; 3647 University of Groningen, the Netherlands; 26656 Ghent University, Belgium; 3647 University of Groningen, the Netherlands

**Keywords:** pupillometry, pupil light reflex, psychosensory pupil reflex, pupil size, luminance, subjective brightness perception

## Abstract

When the pupil dilates, the amount of light that falls onto the retina increases. However, in daily life, this does not make the world look brighter. Here we asked whether pupil size (resulting from active pupil movement) influences subjective brightness in the absence of indirect cues that, in daily life, support brightness constancy. We measured the subjective brightness of a tester stimulus relative to a referent as a function of pupil size during tester presentation. In Experiment 1, we manipulated pupil size through a secondary working-memory task (larger pupils with higher load and after errors). We found some evidence that the tester was perceived as darker, rather than brighter, when pupils were larger. In Experiment 2, we presented a red or blue display (larger pupils following red displays). We again found that the tester was perceived as darker when pupils were larger. We speculate that the visual system takes pupil size into account when making brightness judgments. Finally, we highlight the challenges associated with manipulating pupil size. In summary, the current study (as well as a recent pharmacological study on the same topic by another team) is intriguing first steps towards understanding the role of pupil size in brightness perception.

## Introduction

Our senses adjust their properties in interaction with the environment (Mathôt, 2020). In the case of the visual system, the eyes’ pupils constrict and dilate, thus changing light influx by a factor of about 16 based on the normal variation of pupil diameter between 2 and 8 mm (Lee et al., 2011; Mathôt, 2018; McDougal & Gamlin, 2008). Perception of brightness, even illusory brightness (Laeng & Endestad, 2012; Zavagno et al., 2017), clearly causes changes in pupil size. However, in real life, the opposite does not hold: Changes in pupil size do not make us perceive the world as brighter or darker, despite considerable changes in light influx. This brightness constancy is likely due, at least in part, to many indirect cues in our environment, such as light intensity and knowledge about an object's brightness (e.g., Shevell & Kingdom, 2008). It is still unclear, however, whether and how pupil size affects brightness perception in the absence of such cues.

Although many studies have used pupil size as a physiological marker of arousal and other cognitive factors (Goldwater, 1972; Laeng et al., 2012), far fewer studies have investigated how changes in pupil size affect visual perception (Mathôt, 2018). This question was addressed only in a small series of classic studies (Campbell & Gregory, 1960; Denton, 1956; Woodhouse, 1975), and again in a series of more recent studies (e.g., Ajasse et al., 2018; Bombeke et al., 2016; Mathôt & Ivanov, 2019; Thigpen et al., 2018). For instance, Mathôt and Ivanov (2019) manipulated pupil size by varying the brightness of a stimulus in peripheral vision. They found that small pupils were associated with improved discrimination of detailed stimuli in central vision; in contrast, large pupils were associated with improved detection of faint stimuli outside of central vision. This finding confirms that visual acuity is best when pupils are small, whereas visual sensitivity is best when pupils are large. Findings such as this suggest that one possible function of pupil-size changes is to optimally shift the balance between visual sensitivity and acuity, a trade-off that depends on many cognitive (notably arousal) and environmental (notably brightness and distance) factors (Mathôt, 2020; Mathôt & Van der Stigchel, 2015). More generally, flexible pupil-size changes may help to optimise performance in complex yet efficient ways.

Most recently, and especially relevant to the current study, Sulutvedt et al. (2021) tested whether pupil size affects subjective brightness perception. They treated one eye of each participant with a pharmacological agent (Tropicamide) to induce pupil dilation. During the experiment, a referent stimulus with certain brightness was presented to one eye, while the other eye was covered. Then the eye cover was switched such that participants would see a tester stimulus with the eye that had previously been covered; next, participants adjusted the brightness of the tester to match that of the referent. A perceived-brightness change (i.e., a difference between the perceived brightness of the referent and the adjusted tester) was calculated to inspect how differences in pupil size (between both eyes) resulted in differences in subjective brightness perception. The results demonstrated that, when seen by the dilated eye, the tester had to be darker than the referent to be perceived as equally bright, indicating a brightness overestimation when pupils were large. In other words, in this study, it appeared that the increased light influx associated with pupil dilation resulted, as one might expect, in an increased perception of brightness.

In the current study, we used a different approach to explore the effect of pupil-size changes on subjective brightness perception in the absence of indirect cues. As a crucial departure from Sulutvedt et al. (2021), we did not induce changes in pupil size pharmacologically, but instead used cognitive (Experiment 1) or visual (Experiment 2) factors that are known to affect pupil size. This is a crucial difference, because the visual system is likely unable to make changes in pupil size into account when these are induced pharmacologically, as in Sulutvedt et al. (2021). Whereas, the visual system may be able to do so when pupil-size changes are induced “endogenously,” as in the current study. To make the analogy with eye movements, the study by Sulutvedt et al. (2021) would be similar to moving your eyeball by putting some (mild) pressure on it from the side using your finger, which results in a perceived movement of the world. In contrast, our study would be similar to making a regular eye movement, which does *not* result in a perceived movement of the world, likely because the visual system does not interpret the visual consequences of eye movements as reflecting actual movement “out there” (Mathôt & Theeuwes, 2011; Wurtz, 2018).

We conducted two experiments using two paradigms that allowed us to manipulate participants’ pupil size in different ways. We asked participants to compare the brightness of two stimuli: a referent of a fixed brightness and a tester of a variable brightness. In Experiment 1, we manipulated pupil size by manipulating memory load in a visual working-memory task. Based on many previous studies (e.g., Kahneman & Beatty, 1966), we expected that pupil size in the low-load condition would be smaller than in the high-load condition. In Experiment 2, we manipulated pupil size by presenting red and blue displays. Short-wavelength blue light, as compared to isoluminant red light, is known to induce a strong response of intrinsically photosensitive retinal ganglion cells (ipRGCs), which results in slow-but-sustained pupil constriction (Berson et al., 2002; Gamlin et al., 2007; Gooley et al., 2012). Accordingly, we expected that pupil size in the blue condition would be smaller than in the red condition. In all experiments, we hypothesised that changes in pupil size during the presentation of the tester stimulus would lead to a difference in how the brightness of the tester would be perceived relative to that of the referent. However, we did not have a specific prediction about the direction of the effect, that is, whether pupil dilation would consistently lead to an overestimation (cf., Sulutvedt et al., 2021) or underestimation of subjective brightness.

In our analyses, we will focus on the contrast between conditions (e.g., whether the perceived brightness of the tester depends on the colour of the preceding display in Experiment 2), rather than on the difference in pupil size during the presentation of the referent and the tester. Phrased differently, we do not focus on whether the perceived brightness of the tester depends on the relative size of the pupil during the presentation of the tester as compared to the referent. The rationale behind this is that all conditions may be affected by a systematic overestimation or underestimation of the tester brightness that is unrelated to pupil size, in which case the most valid comparisons are between those conditions. Throughout this article, we use the term “luminance” to refer to the physical stimulus and “brightness” to refer to the psychological perception as experienced by a participant.

### Data Acquisition, Processing, and Analyses

All experiments were approved by the Ethical Committee of Psychology at the University of Groningen (Reference no.: 17012-S-NE). Participants were recruited via the university's participant pool. Each experiment in this study had different samples of participants, naïve to the aim of the experiment. Participant's consent was collected prior to the experiment and participation was compensated with course credit. The experiments were conducted in the same room with a constant, low level of ambient luminance (<1 lux). Sources of light were the eye-tracker, the display of the eye-tracking system, and the stimulus-presentation display; other light sources in the room were turned off. Participants were asked to sit in the room for at least 5 minutes before starting the experiment.

Experiments were conducted with OpenSesame (Mathôt et al., 2012) using PyGaze for eye tracking (Dalmaijer et al., 2014). Eye position and pupil size were recorded monocularly using the EyeLink 1000 + Desktop Mount eye-tracker (SR Research, Ontario, Canada) at a sampling rate of 1000 Hz. A head support was used for participants to remain in a stable posture and to keep approximately a 40 cm distance between the computer screen and the cornea. Participants were debriefed at the end of each experiment.

Prior to further processing, we downsampled the pupil data to 100 Hz and reconstructed eye blinks using the cubic–spline interpolation (Mathôt, 2013). After that, we converted pupil area (in pixels) to pupil diameter (in millimetres), using a conversion formula that we had previously determined for our setup (Mathôt & Ivanov, 2019). A trial-by-trial baseline correction was performed by subtracting the median pupil size during the baseline period from the entire pupil waveform during the epoch of interest (see the “Method” subsection under the “Experiment 1” section and the “Method” subsection under the “Experiments 2a and 2b” section). No correction for eye-movement artefacts was done in the preprocessing stage.

In Experiments 2a and 2b (see the subsection “Design, Materials, and Procedure”), we performed an isoluminant calibration to find intensities of red and blue that lead to an equally strong short-term pupil constriction (while the long-term pupil constriction, driven by ipRGCs remains the strongest in response to blue). To test whether this calibration procedure was successful, we verified that the pupil constriction in response to the last two blue displays was equal in magnitude to that in response to the last two red displays.

Participants’ subjective perception of the brightness of the tester stimulus (see the subsection “Method” under the section “Experiment 1” and subsection “Method” under the section “Experiments 2a and 2b”) was fitted with a sigmoid curve using a logistic function. From this function, we obtained the individual *k* parameters representing the steepness of the curve along the *y*-axis and the *x*_0_ parameters representing the sigmoid's midpoint along the *x*-axis. In this sigmoid curve, the *y*-axis represented participants’ brightness judgment of the tester, ranging from 0 (always darker than the referent) to 1 (always brighter than the referent) and the *x*-axis represented the luminance levels of the tester stimulus ranging from the darkest to the brightest. A rightward shift of the sigmoid's midpoint would indicate that the tester stimulus had to be brighter to be judged equally bright as the referent stimulus (i.e., a luminance underestimation). Conversely, a leftward shift of the sigmoid's midpoint would indicate that the tester stimulus had to be darker to be judged equally bright as the referent stimulus (i.e., a luminance overestimation). We used a least-squares method for the statistical analysis of the curve fitting.

Pupil-size changes over time will be shown in the “Results” section, but we statistically analysed the data using the aggregated median (*Md*) values of the baseline-corrected pupil data in the epoch of interest and the mean (*M*) values of luminance judgment. To aggregate the pupil data, we first aggregated the pupil samples across time points in each trial using the median. Then, separately in each condition, we calculated the median pupil size across trials per participant. Finally, we have the median value of pupil size per participant in each condition. We used JASP (JASP Team, 2020) for performing separate Bayesian paired-samples Student *t*-tests to compare the pupil size and brightness perception between two conditions. In all our statistical analyses, the default value of 0.707 for the Cauchy distribution was used as our priors. BF_10_ > 1 would indicate support for the alternative model; meanwhile, BF_01_ > 1 would indicate support for the null model. We used Jeffreys’ qualitative descriptions to interpret the resulting Bayes factors (Jarosz & Wiley, 2014; Wetzels et al., 2011). Our experimental tasks, data files, statistical analyses, as well as the processing pipelines are available on https://osf.io/ynsmf/.

## Experiment 1

**Figure 1.**
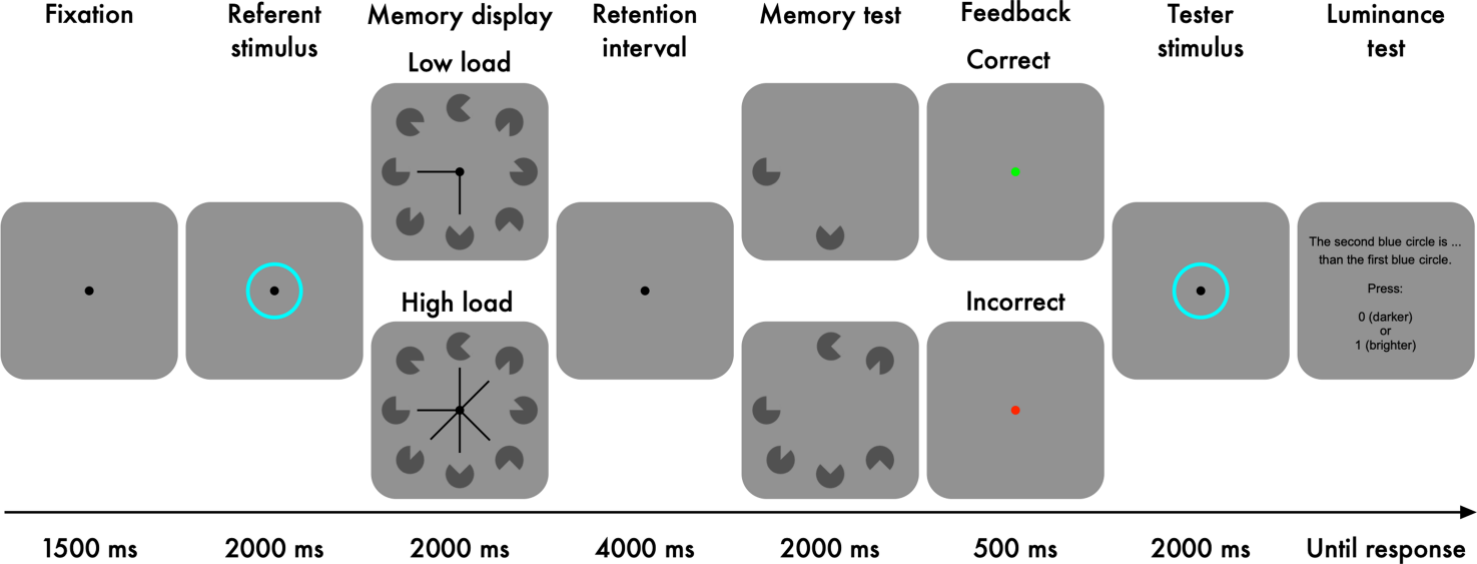
Experimental task of Experiment 1.

In Experiment 1, participants indicated whether a tester stimulus was brighter or darker than a previously presented referent stimulus (Figure 1). To manipulate pupil size, participants performed a visual-working-memory task in between the presentation of the referent and the tester stimuli; we varied the working-memory load, because previous studies have shown that the pupil dilates with increasing working-memory load (Kahneman & Beatty, 1966; Unsworth & Robison, 2015).

A referent stimulus was presented at the beginning of each trial, then pupil size was manipulated using a low- and high-load memory task. Feedback was given immediately after every response (or after a timeout) on the memory test. Judgment of the luminance level of the tester stimulus was done at the end of the trial.

### Method

#### Participants

Thirty observers participated in the experiment (*M*_age_ = 19.93 years, *SD* = 1.77, age-range = 18–27 years, 23 women). Participants had normal or corrected-to-normal visual acuity.

#### Design, Materials, and Procedure

Each trial started with a fixation dot presented for 1500 ms (Figure 1). *Luminance referent*: Next, a referent stimulus, an aqua-coloured ring, was displayed for 2000 ms. The luminance level of the referent stimulus was held constant across trials and participants. We used this epoch as our baseline period. *Memory task*: Next, eight Pacman-like items with various rotations were presented for 2000 ms. A central cue, consisting of line segments, pointed at either two (low load) or six (high load) items, and participants were instructed to memorise only the cued items (i.e., targets). Following the memory display, there was a retention interval during which a fixation dot was presented for 4000 ms. After that, the targets were presented again until a response was given with a timeout of 2000 ms. In 50% of the trials, one of the targets was rotated differently from the original, and participants indicated whether there was a change or not.

A green dot (Hue-Saturation-Value: 120°, 100%, 100%) or a red dot (Hue-Saturation-Value: 0°, 100%, 100%) was shown for 500 ms after a correct response or incorrect response, respectively. *Luminance tester*: Next, the tester stimulus, another aqua-coloured ring, was displayed for 2000 ms. The luminance level of the tester was varied relative to that of the referent (Table 1). Finally, participants indicated whether the tester was darker or brighter than the referent. Stimuli were presented on a grey background (2.84 cd/m^2^) with a resolution of 1920  ×  1080 pixels (27′′ flat screen Iiyama monitor).

**Table 1.** Chromaticity (*X*, *Y*, *Z*) and luminance (in cd/m^2^) of the stimuli in all experiments.

Stimulus	Level	Experiment 1	Experiments 2a and 2b
Referent stimulus	Isoluminant with tester	(115, 159, 226); 13.6	(16, 8, 33); 0.6
Tester stimulus	Darkest	(102, 134, 254); 11.4	(9, 4, 18); 0.4
	Darker than referent	(111, 151, 255); 12.9	(12, 6, 26): 0.5
	Isoluminant with a referent	(115, 159, 226); 13.6	(16, 8, 33); 0.6
	Brighter than a referent	(117, 164, 256); 13.9	(20, 9, 41); 0.8
	Brightest	(125, 178, 257); 15.2	(26, 11, 52); 1.0

We used a 2 (memory load: low vs. high)  ×  5 (luminance level: darkest vs. darker vs. isoluminant vs. brighter vs. brightest) within-participant design. The task started with five practice trials followed by 200 fully randomised experimental trials. The experimental trials were divided into 40 trials  ×  5 blocks with a break in between the blocks.

### Results

#### Pupil Size Difference by Memory Load

**Figure 2.**
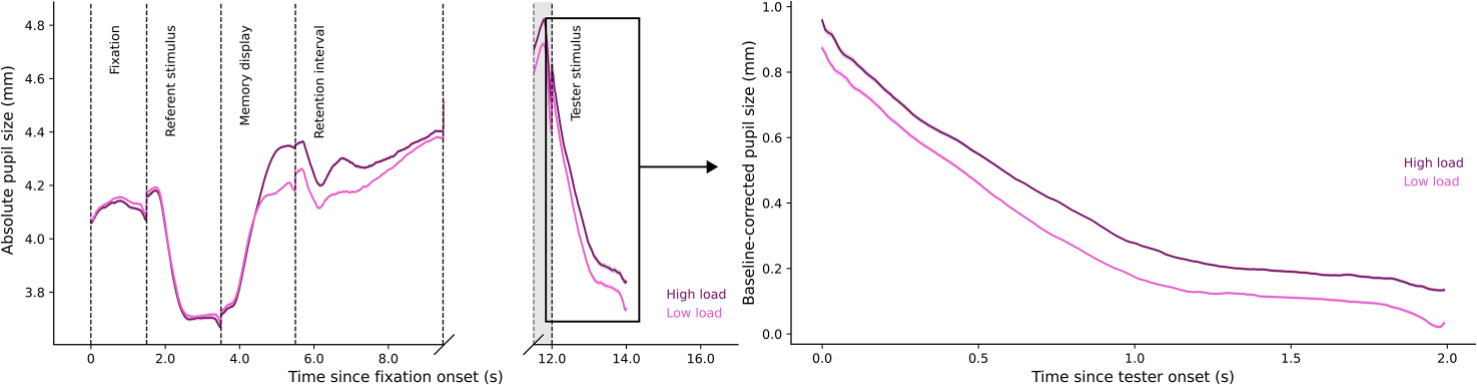
Plots of pupil size by memory load.

Figure 2 (left panel) shows pupil size modulations for the whole trial. We compared pupil size in the tester stimulus phase between low load and high load conditions. The resulting Bayes factor, BF_10_ = 22386.86, indicates extremely decisive evidence for an effect of memory load on pupil size. As shown in Figure 2 (right panel), pupil size in the low load condition (*Md* = 0.258, *SD* = 0.178) was smaller than pupil size in the high load condition (*Md* = 0.328, *SD* = 0.192).

Left panel: Absolute pupil size across the entire trial sequence. The vertical dashed lines indicate the onset of the stated phase in each trial. The shaded grey area indicates the feedback phase. The broken *x*-axis with diagonal lines indicates the memory test phase during which response times varied. Right panel: Pupil size shown here is stimulus-locked to the tester stimulus phase and baseline-corrected to the referent stimulus phase. There was very strong evidence that pupil sizes in the two conditions were different. The coloured shadings around the trace of the pupil size, although very narrow, indicate a 95% within-subject confidence interval.

#### Brightness Judgment Difference by Memory Load

**Figure 3.**
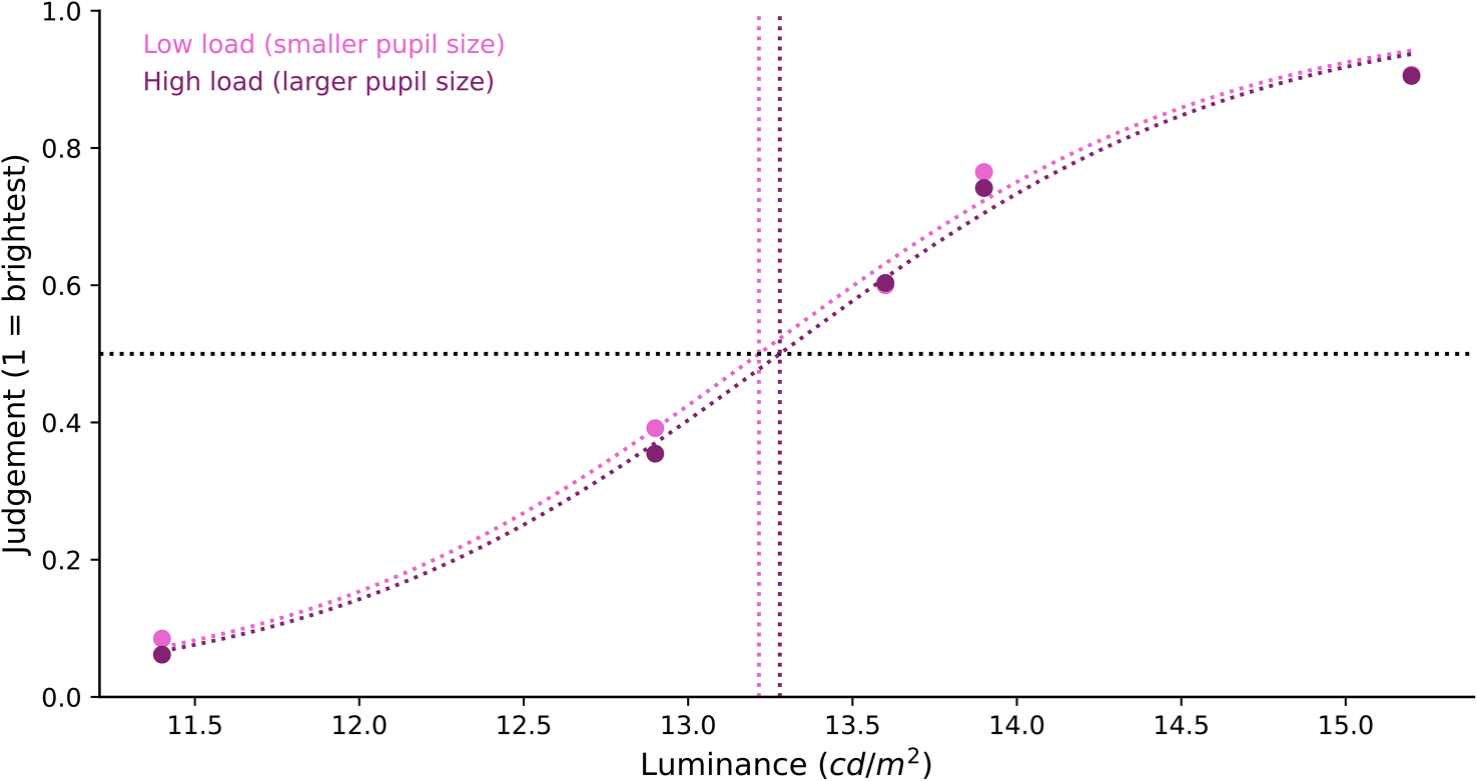
Brightness judgment differences by memory load. Participants’ subjective brightness judgment of the tester stimulus was fitted with a sigmoid curve. We found no difference in the brightness judgment between the low- and high-load conditions.

To analyse the brightness judgment, we compared the *x*_0_ values in the low-load and high-load conditions. The difference in the sigmoid's midpoint shifts between the low-load (*M_x_*_0_ = 13.264, *SD* = 0.538) and high-load (*M_x_*_0_ = 13.333, *SD* = 0.444) conditions (Figure 3) was small. The corresponding estimated Bayes factor (BF_10_ = 0.333) provided anecdotal evidence in favour of the null hypothesis for no difference. In other words, and in contrast to our prediction, a slight change in pupil size as induced by our working-memory-load manipulation did not appear to induce a notable change in perceived brightness. To put this in context, in the absence of any brightness constancy, we would have expected a rightwards shift of the midpoint of 1.9% (based on the amount of light that enters the eye, given the difference in pupil size between the high- and low-memory load conditions).

##### Pupil Size Difference by Performance Feedback

**Figure 4.**
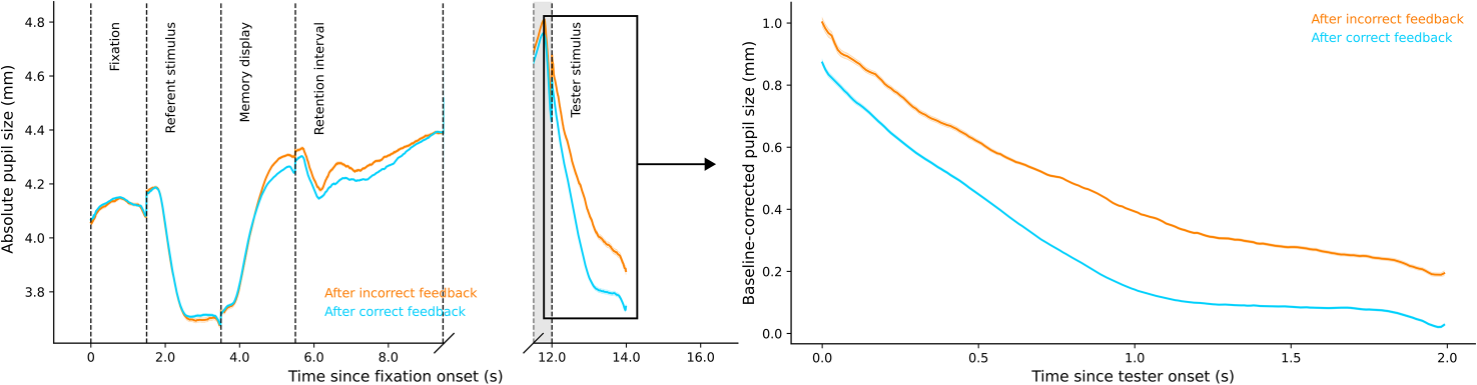
Plots of pupil size by performance feedback.

In a post hoc analysis, we also found decisive evidence, BF_10_ = 5.421×10^6^, for pupil-size differences during the tester stimulus phase after correct versus incorrect performance feedback (Figure 4, right panel). Pupil size after correct feedback (*Md* = 0.218, *SD* = 0.172) was smaller than pupil size after incorrect feedback (*Md* = 0.478, *SD* = 0.214). Strikingly, the difference in pupil size between correct (66.36% of trials) and incorrect trials was considerably larger than the difference between low- and high-load trials.

Left panel: Absolute pupil size across the entire trial sequence. The vertical dashed lines indicate the onset of the stated phase in each trial. The shaded grey area indicates the feedback phase. The broken *x*-axis with diagonal lines indicates the memory test phase during which response times varied. Right panel: Pupil size shown here is stimulus-locked to the tester stimulus phase and baseline-corrected to the referent stimulus phase. We observed smaller pupils after correct feedback relative to after incorrect feedback. The coloured shadings around the trace of the pupil size, although very narrow, indicate a 95% within-subject confidence interval.

##### Brightness Judgment Difference by Performance Feedback

**Figure 5.**
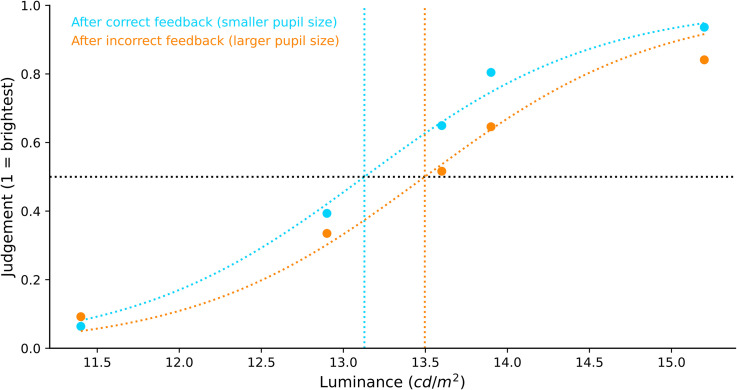
Brightness judgment by performance feedback.

Due to the association between performance feedback and pupil size during the tester stimulus phase, we also analysed participants’ brightness judgments after correct and incorrect responses. Participants’ subjective brightness perception was again fitted with a sigmoid curve using the logistic function as before. We performed a Bayesian paired-samples *t*-test to compare the *x*_0_ values after correct and incorrect responses, and found very strong evidence for an effect of response accuracy on brightness judgments, BF_10_ = 76.627. As seen in Figure 5, the sigmoid's midpoint of the correct responses (*M_x_*_0_ = 13.185; *SD* = 0.498) shifted to the left compared to the incorrect responses (*M_x_*_0_ = 13.530; *SD* = 0.549), indicating that there was a luminance overestimation of the tester stimulus when pupils were small as compared to large. To put this in context, in the absence of any brightness constancy, we would have expected a rightwards shift of the midpoint of 5.4% (based on the amount of light that enters the eye, given the difference in pupil size between incorrect and correct responses). With perfect brightness constancy, we would have expected no shift of the midpoint; however, we in fact observed a leftwards shift of 2.3%.

Participants’ subjective brightness perception of the tester stimulus was fitted with a sigmoid curve. We found that the luminance of the tester stimulus was overestimated when pupils were small (after correct feedback) as compared to large (after incorrect feedback).

### Discussion

In Experiment 1, we manipulated pupil size using memory load in a visual working-memory task. In line with previous studies (Kahneman & Beatty, 1966), we found that pupil size was smaller in the low-load condition than in the high load condition. Furthermore, we hypothesised that participants would perceive the luminance of the tester stimulus differently depending on the size of their pupils, as manipulated through memory load. However, our primary analysis did not support this hypothesis.

Additionally, we found a strong relationship between performance feedback and pupil size, such that pupil size after incorrect feedback was larger than pupil size after correct feedback. This was accompanied by an effect on perceived brightness, such that participants underestimated the brightness of the tester when their pupils were large (after incorrect feedback), as compared to when their pupils were small (after correct feedback).

Possibly, the effect of memory load on pupil size was too small to result in an effect on perceived brightness. In contrast, the difference between pupil size after incorrect and correct responses was far larger (as shown in the section “Results Summary”), and here we did find a corresponding effect on perceived brightness. This result is suggestive, but it is conceivable that other, non-pupil-size-related factors play a role as well. Therefore, we conducted the second experiment using a different way to manipulate pupil size, with the aim to find converging evidence from different approaches for an association between pupil size and perceived brightness.

## Experiments 2a and 2b

In Experiments 2a and 2b, to manipulate pupil size, we used the fact that the retina contains ipRGCs that are primarily activated by short-wavelength (∼470 nm) blue stimuli (Mathôt, 2018; McDougal & Gamlin, 2008). Activation of ipRGCs causes a prolonged pupil constriction after several seconds. (The initial pupil constriction on which we based our isoluminance-calibration procedure, described in more detail below, is driven by rods and cones). Therefore, we can manipulate pupil size by presenting blue stimuli (small pupils) or red stimuli (large pupils) in between the presentation of the referent and the tester.

The second experiment consisted of two sub-experiments. In Experiment 2a, there was a programming issue in the procedure to determine isoluminant shades of red and blue. Therefore, we conducted Experiment 2b with a corrected and improved isoluminance calibration. However, although we felt that it was prudent to replicate the results of Experiment 2a with an improved procedure, the issue did not notably affect the calibration results nor the main results, and we therefore present the results of both sub-experiments here.

In Experiments 2a and 2b, as compared to Experiment 1, we used lower brightness intensities for the referent and tester stimuli, and a smaller relative difference between the darkest and the brightest referent stimulus. This should make the brightness-judgment task more difficult, thus potentially allowing us to pick up more subtle effects.

### Method

#### Participants

Participants had normal or corrected-to-normal visual acuity. In Experiment 2a, 30 naïve participants volunteered in the experiment (*M*_age_ = 20.23 years, *SD* = 3.33, age-range = 18–34 years, 27 women). In Experiment 2b, 16 new participants volunteered in the experiment (*M*_age_ = 21.6 years, *SD* = 3.32, age-range = 18–32 years, 10 women).

#### Design, Materials, and Procedure

The design, materials, and procedures in Experiments 2a and 2b were the same. Both experiments consisted of two parts: an isoluminance calibration and an experimental task.

**Figure 6.**
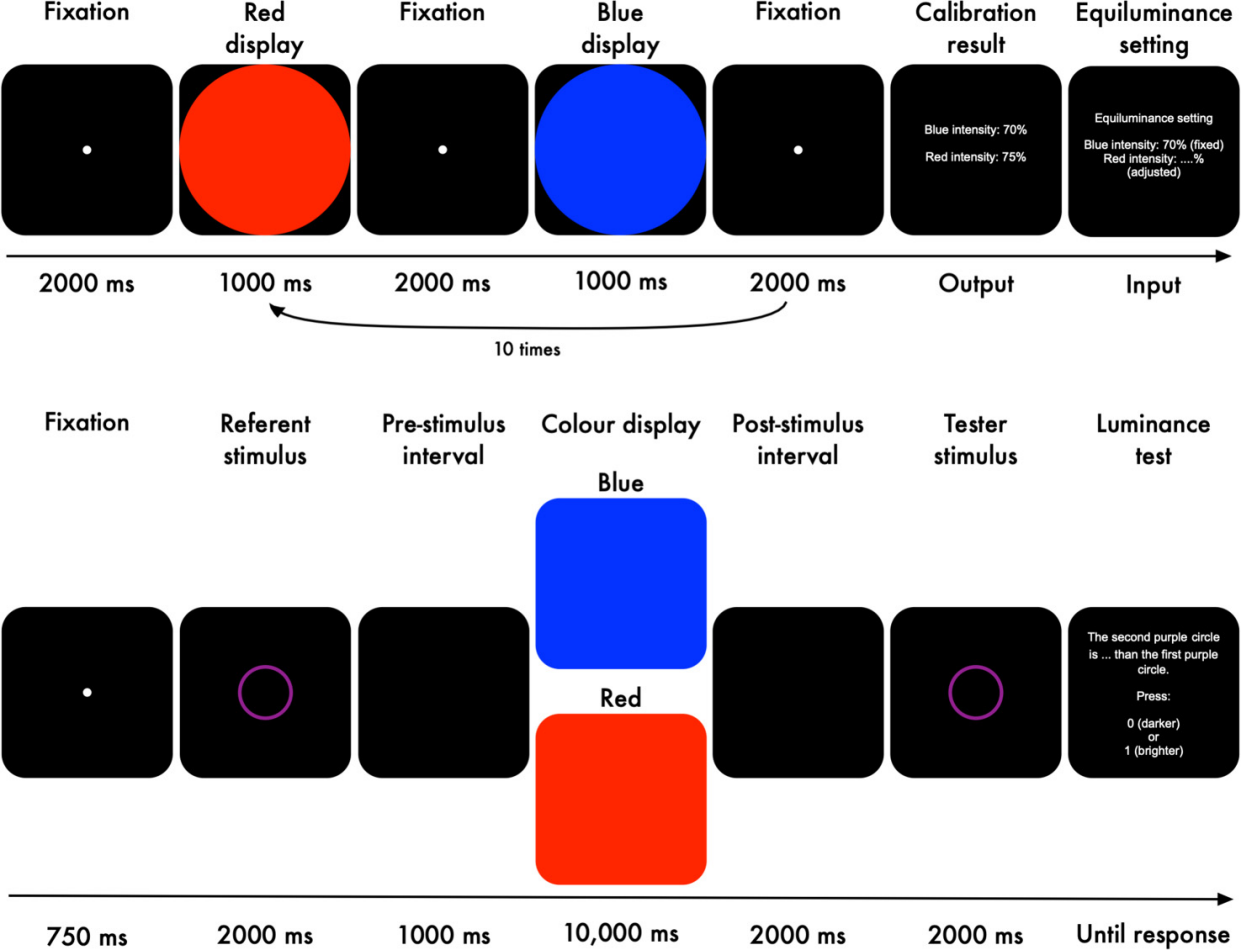
Isoluminance calibration and the experimental task of Experiments 2a and 2b.

*Isoluminance Calibration.* We calibrated the red and blue for each participant to be isoluminant in the specific sense described below. Each trial started with a fixation of 2000 ms (Figure 6, top panel). *Colour display*: Next, a red or a blue circle was presented on a black background for 1000 ms. Participants fixated on the centre of the screen during the colour presentation. After that, a fixation dot was presented again for 2000 ms. The second colour display, with a colour that was different from the previous one, was presented for 1000 ms, followed by a fixation dot for 2000 ms. This cycle repeated until each colour was presented ten times. *Intensity level*: We made the two colour displays isoluminant by iteratively adjusting the intensity level of the red display (the intensity of the blue display was fixed) based on the strength of the initial pupil constriction following the display onset until both colours triggered an equally strong pupil constriction. The strength of pupil constriction was determined online as the difference between the largest and smallest pupil size (smoothed but not baseline-corrected) after display onset. Specifically, when a blue display triggered a stronger pupil constriction than the preceding red display, the intensity of the red display was increased by 1% (in red–green–blue units); otherwise, its intensity was decreased by 1%. Conversely, when a red display triggered a stronger pupil constriction than the preceding blue display, the red intensity was decreased; otherwise, its intensity was increased. This calibration procedure consisted of 10 red displays and 10 blue displays in Experiment 2a, and 20 red displays and 20 blue displays in Experiment 2b. Importantly, even though we calibrated our colours based on the initial pupil constriction, the long-term pupil constriction, which is driven largely by ipRGCs, was still more pronounced in response to blue displays (Mathôt, 2018; McDougal & Gamlin, 2008).

Top panel: The intensity of the red and blue displays was calibrated such that both displays triggered initial pupil constrictions of the same magnitude. Bottom panel: A referent stimulus was presented at the beginning of the trial. The calibrated blue or red display was used to manipulate participants’ pupil size. Participants judged the luminance of the tester stimulus after the manipulation.

*Experimental Task.* Each trial started with a fixation dot presented for 750 ms (Figure 6, bottom panel). *Luminance referent*: Next, a referent stimulus, a purple ring, was displayed for 2000 ms. The colour purple was selected as a combination of (or equally similar to) the red and blue colours that were used to manipulate pupil size. The luminance level of the referent stimulus was held constant across trials and participants. We used this epoch as our baseline period. *Pre-stimulus interval*: An empty black screen was shown for 1000 ms as an interval prior to the colour stimuli. *Colour stimuli*: We used the calibrated red and blue displays to manipulate pupil size. The colours were presented on the whole screen for 10,000 ms; this duration was long enough for the influence of ipRGCs to arise, thus resulting in a difference in pupil size between the red and blue displays. *Post-stimulus interval*: Another empty black screen was shown for 2000 ms after the colour stimuli. *Luminance Tester*: Next, the tester stimulus, another purple ring, was displayed for 2000 ms. The luminance level of the tester was varied relative to that of the referent stimulus (Table 1). Finally, participants indicated whether the tester was darker or brighter than the referent. Stimuli were presented on a black background (0.16 cd/m^2^) with a resolution of 1920  ×  1080 px (27′′ flat screen Iiyama monitor).

We used a 2 (isoluminant colour: red vs. blue)  ×  5 (luminance level: darkest vs. darker vs. isoluminant vs. brighter vs. brightest) within-participant design. The isoluminance calibration comprised 20 trials (i.e., 10 trials for each of the colour stimuli). The experimental task started with two practice trials followed by 140 fully randomised experimental trials for each participant. The experimental trials were divided into 20 trials  ×  7 blocks with a break in-between the blocks.

### Results

#### Isoluminance Calibration

**Figure 7.**
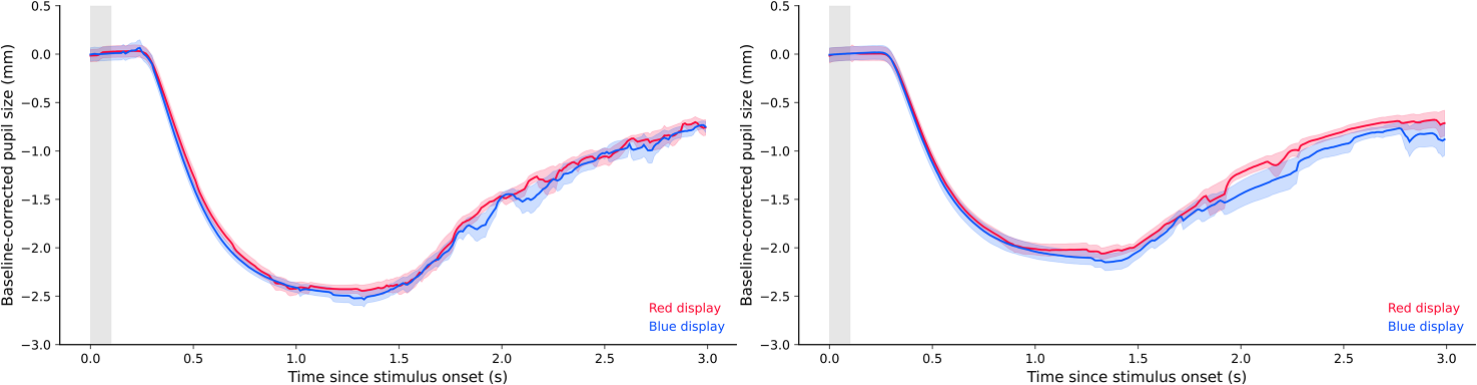
Plots of calibrated pupil size in Experiments 2a and 2b.

*Experiment 2a*. After isoluminance calibration, the mean chromaticity coordinates of the red stimulus were 0.63 (*x*), 0.33 (*y*), and 31.92 (cd/m^2^, *Y*); the chromaticity coordinates of the blue stimulus were 0.16 (*x*), 0.05 (*y*), and 7.66 (cd/m^2^, *Y*). Figure 7 (left panel) depicts the pupil responses to the red and blue displays at the end of the isoluminance calibration. We performed a baseline correction using the first 100 ms of the stimulus presentation. As described in Section “Data Acquisition, Processing, and Analyses,” we analysed only the last two trials of each colour to see if the calibration procedure had properly converged. We compared median pupil size during display presentation as a dependent variable, and colour condition as an independent variable. This showed anecdotal support for the null model, meaning that there was some support for equivalent pupil constrictions in the red and blue conditions, BF_01_ = 2.306 (Figure 7, left panel). In other words, despite a programming error in the procedure (which is why we conducted Experiment 2b), the outcome was still a satisfying calibration. Importantly, differences in luminance, even slight differences, have a very strong effect on pupil constriction. Therefore, even though it was difficult to establish perfect equivalence with our limited number of observations (two pupil responses per colour per participant), it seemed that any imperfections in the calibration results were minor.

Pupil size following display onset during isoluminance calibration (last two displays of each colour only). The grey area shows the baseline phase. Results showed anecdotal support for the null model in both experiments, that is, pupil responses to the red and blue displays during the isoluminance calibration were not different. Coloured shadings around the trace of the pupil size represent a 95% within-subject confidence interval. Left panel: Experiment 2a; right panel: Experiment 2b.

*Experiment 2b*. After isoluminance calibration, the mean chromaticity coordinates of the red stimulus were 0.63 (*x*), 0.33 (*y*), and 33.34 (cd/m^2^, *Y*); the chromaticity coordinates of the blue stimulus were 0.16 (*x*), 0.05 (*y*), and 7.66 (cd/m^2^, *Y*). We performed the same statistical analysis as in Experiment 2a, and again found anecdotal evidence for the null model, indicating again that pupil constrictions were roughly equal in the red and blue conditions, BF_01_ = 1.498 (Figure 7, right panel).

#### Pupil Size During Tester Presentation as a Function of Display Colour

**Figure 8.**
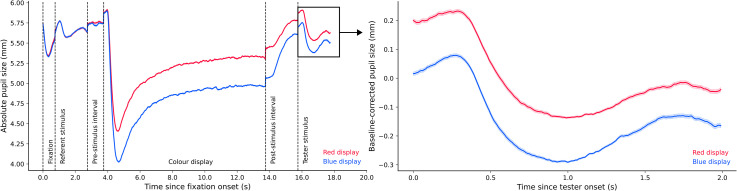
Plots of experimental pupil size in Experiment 2a.

*Experiment 2a*. Figure 8 (left panel) shows pupil size over time for the entire trial sequence. We compared median pupil size during tester presentation between the red and blue conditions. We found extremely decisive evidence for an effect of colour condition on pupil size, BF_10_ = 2.045 × 10^6^. As can be seen in Figure 8 (right panel), pupil size in the blue condition (*Md* = −0.114, *SD* = 0.219) was smaller than pupil size in the red condition (*Md* = 0.007, *SD* = 0.192).

Left panel: Absolute pupil size across the entire trial sequence. The vertical dashed lines indicate the onset of the stated phase in each trial. Right panel: Pupil size shown here was stimulus-locked to the tester stimulus phase and baseline-corrected to the referent stimulus phase. There was extremely decisive evidence that pupil response to the red display was larger than that to the blue display. The coloured shadings around the trace of the pupil size, although very narrow, indicate a 95% within-subject confidence interval.

**Figure 9.**
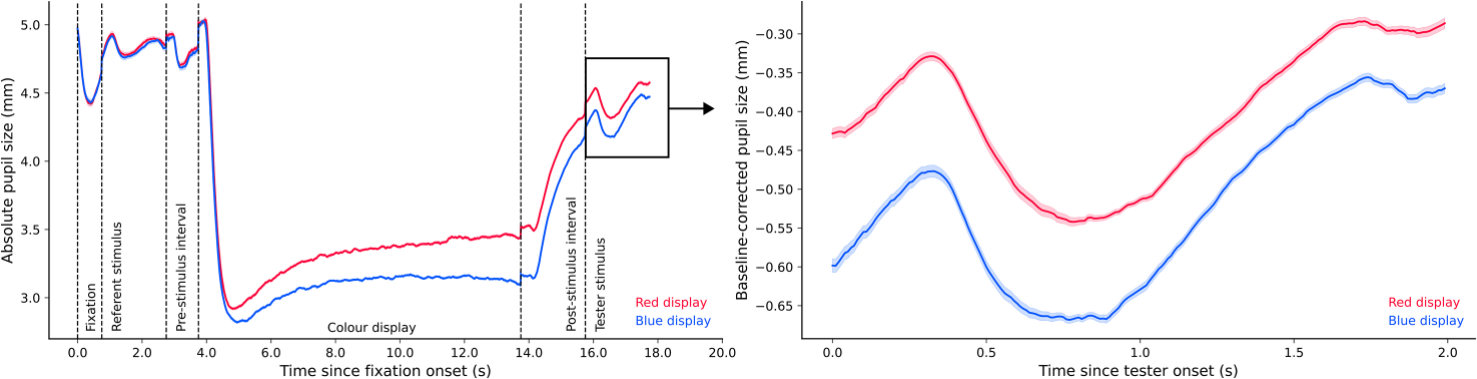
Plots of experimental pupil size in Experiment 2b.

*Experiment 2b*. Figure 9 (left panel) shows pupil size over time for the entire trial sequence. The analysis steps were the same as those in Experiment 2a. The resulting Bayes factor indicated very strong evidence for the alternative model relative to the null model, BF_10_ = 27.77. Figure 9 (right panel) shows that pupil size in the blue condition (*Md* = −0.515, *SD* = 0.252) was smaller than pupil size in the red condition (*Md* = −0.321, *SD* = 0.237).

Left panel: Absolute pupil size across the entire trial sequence. The vertical dashed lines indicate the onset of the stated phase in each trial. Right panel: Pupil size shown here is stimulus-locked to the tester stimulus phase and baseline-corrected to the referent stimulus phase. There was extremely decisive evidence that pupil response to the red display was larger than that to the blue display. The coloured shadings around the trace of the pupil size, although very narrow, indicate a 95% within-subject confidence interval.

#### Brightness Judgment as a Function of Display Colour

**Figure 10.**
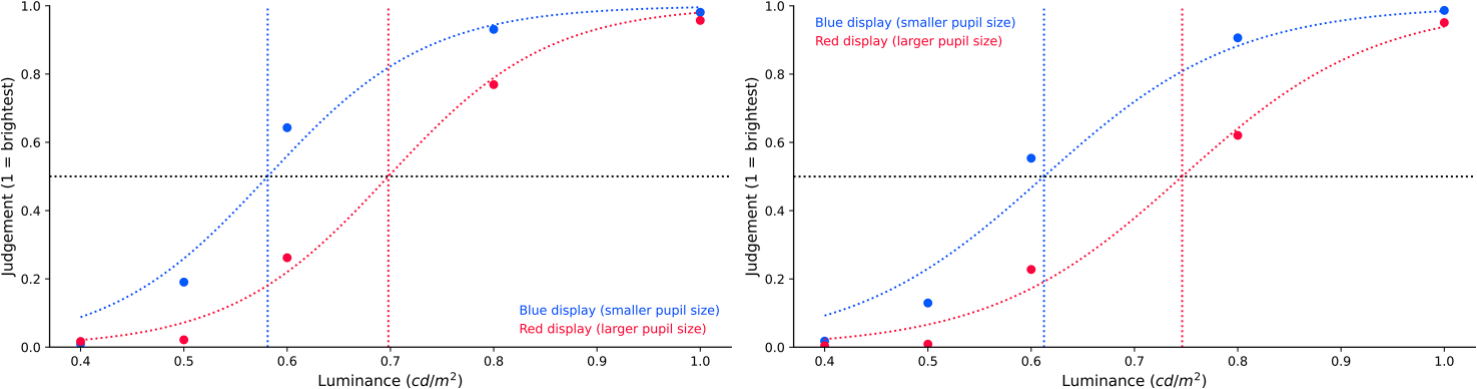
Brightness judgment as a function of display colour.

*Experiment 2a*. To analyse the brightness judgments, we fitted sigmoid curves to the brightness estimations, separately for the blue and red conditions, and compared the *x*_0_ values. We found extremely decisive evidence for an effect of display colour on brightness judgments, BF_10_ = 178565.872. As shown in Figure 10 (left panel), the sigmoid's midpoint in the blue condition (*M_x_*_0_ = 0.585, *SD* = 0.044) shifted to the left, compared to the one in the red condition (*M_x_*_0_ = 0.702, *SD* = 0.078). This indicates that the luminance of the tester stimulus was overestimated when pupils were small (in blue condition) compared to when pupils were large (in red condition).

Participants’ subjective brightness perception of the tester stimulus was fitted with a sigmoid curve. The luminance of the tester stimulus was overestimated when pupils were small (blue condition) as compared to large (red condition). Left panel: brightness perception in Experiment 2a; right panel: brightness perception in Experiment 2b.

*Experiment 2b*. The analysis steps were the same as those in Experiment 2a. We again found strong evidence for an effect of display colour on brightness judgments, BF_10_ = 24.748. As shown in Figure 10 (right panel), the sigmoid's midpoint in the blue condition (*M_x_*_0_ = 0.616, *SD* = 0.076) shifted to the left, compared to the one in the red condition (*M_x_*_0_ = 0.745, *SD* = 0.098). As in Experiment 2a, this indicates that the luminance of the tester stimulus was overestimated when pupils were small (in the blue condition) as compared to when pupils were large (in the red condition).

To put this in context, in the absence of any brightness constancy, we would have expected a rightwards shift of the midpoint of 2.6% (Experiment 2a)/2.9% (Experiment 2b; based on the amount of light that enters the eye, given the difference in pupil size between red and blue conditions). With perfect brightness constancy, we would have expected no shift of the midpoint; however, we in fact observed a leftwards shift of 19.2% (Experiment 2a)/20.1% (Experiment 2b).

### Discussion

In Experiments 2a and 2b, we looked at participants’ subjective brightness perception when pupil size was manipulated using blue and red displays. We first calibrated the intensity of the blue and red colours such that both colours were isoluminant in the sense that they triggered initial pupil constrictions of approximately the same magnitude, after which we used these calibrated colour intensities for the main experiment.

Isoluminance calibration was successful in both experiments (despite a programming issue in Experiment 2a) such that there was no difference in the initial pupil constriction in response to the blue and red displays. However, at later points in time (when the influence of ipRGCs becomes apparent), we found that pupil size in the blue condition was considerably smaller than pupil size in the red condition, and this difference persisted even after the offset of coloured displays until the moment that the tester stimulus was presented. In other words, our manipulation of pupil size was successful.

Crucially, we hypothesised that the brightness of the tester stimulus would be perceived differently as a function of pupil size. Our results in both sub-experiments showed extremely strong evidence that supported this hypothesis, and the direction of the effect was the same as we had found in Experiment 1 for response accuracy. When pupil size was large (in the red condition), the luminance of the tester stimulus was estimated to be lower than when pupil size was small (in the blue condition); that is, in the red condition as compared to the blue condition, the tester had to be brighter to be perceived as equally bright as the referent stimulus.

## Results Summary

**Figure 11.**
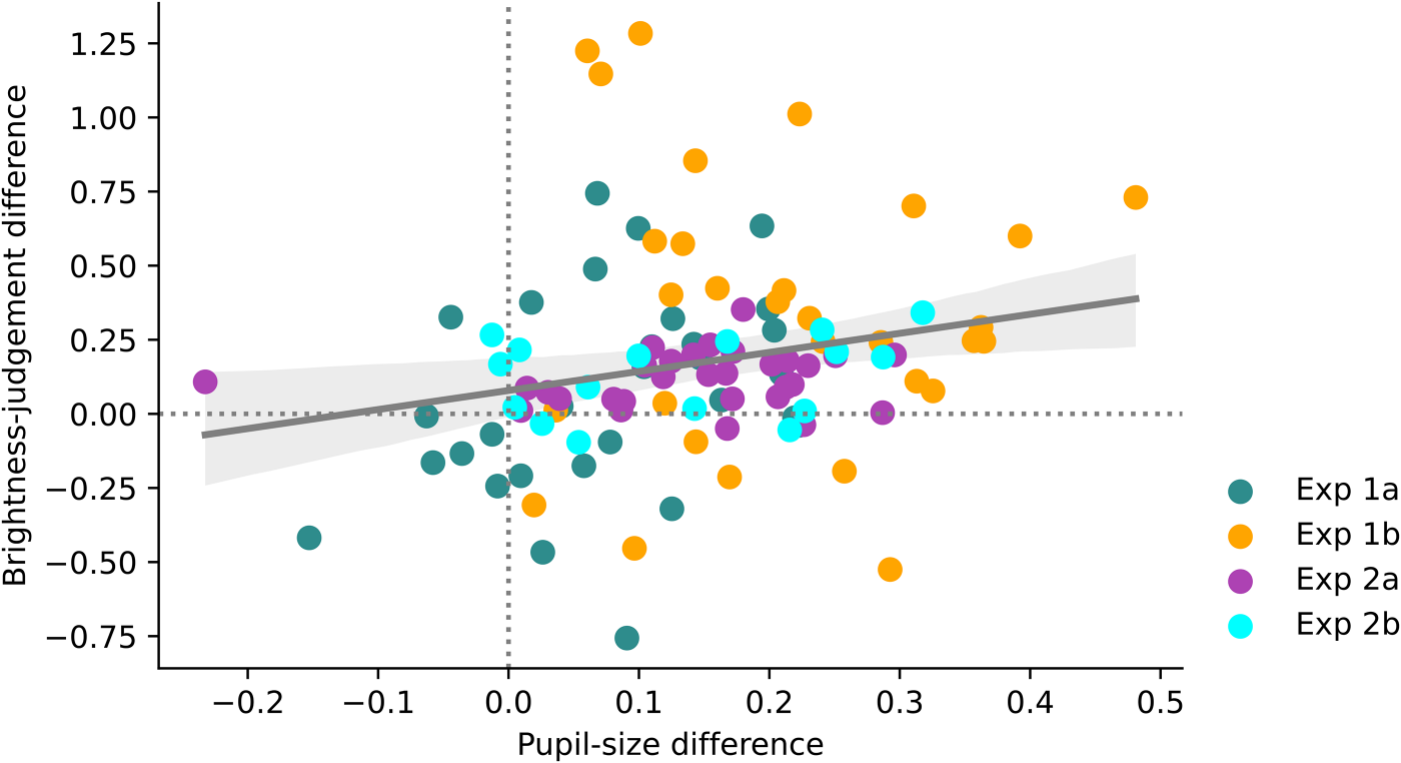
Results summary.

The findings from all experiments are summarised in Figure 11. Most observations are in the upper-right quadrant of the figure, meaning that in most cases an increase in pupil size was associated with a decrease in perceived brightness. However, there was only anecdotal evidence for a between-subject correlation between the pupil-size effect and the brightness judgment (Pearson's *r* = .23, BF_10_ = 1.758).

The difference in perceived brightness (*y*-axis) is a function of the difference in pupil size (*x*-axis) for all experiments and outcome measures. Pearson's correlation coefficient shows *r* = .23, indicating no correlation between pupil-size and brightness-judgment differences.

## General Discussion

In the present study, we explored the effect of pupil size on subjective brightness perception. Specifically, we asked whether changes in pupil size influence the perceived brightness of a stimulus in the absence of indirect cues from our surroundings. To answer this question, we presented participants with a referent stimulus of constant brightness. We then manipulated pupil size with a working-memory task (Experiment 1) or a red/blue colour display (Experiment 2). After the manipulation, we presented a tester stimulus that varied in brightness from trial to trial. We tested participants’ subjective brightness perception by asking them to indicate whether the tester stimulus was brighter or darker than the referent stimulus.

In Experiment 1, we varied the memory load in a working-memory task to manipulate pupil size. In line with previous studies (e.g., Kahneman & Beatty, 1966), we found that pupil size in the low-load condition was slightly smaller than pupil size in the high-load condition. However, we did not observe a difference in brightness perception of the tester stimulus as a function of this pupil-size change. Possibly, the difference in pupil size induced by our memory-load manipulation was simply too small to induce a measurable effect on subjective brightness.

In Experiment 1, we also found that pupil size during the tester stimulus was larger after incorrect responses than after correct responses, a difference that was larger than that induced by memory load. It was very likely that negative feedback or prediction error increased emotional arousal, thus resulting in larger pupil size (e.g., Bradley et al., 2008; Braem et al., 2015; Vanderhasselt et al., 2015). More interestingly, we now also found that pupil dilation after incorrect responses was associated with an underestimation of the brightness of the tester. Clearly, this is a post-hoc finding, and it is conceivable that it was caused by something other than a change in pupil size, such as the visual difference between the red and the green feedback dot (although these were small and roughly isoluminant) or high-level cognitive processes. Nevertheless, the fact that there was an association between pupil size and subjective brightness perception was suggestive and triggered us to conduct Experiment 2, in which we used a more robust method to manipulate pupil size.

In Experiment 2, we exploited the fact that blue light activates ipRGCs more strongly than red light does, which in turn results in a prolonged pupil constriction after exposure to blue stimuli as compared to red stimuli (e.g., Mathôt, 2018). Prior to running the main experiment, we adjusted the brightness of the red and blue stimuli for each participant separately such that both stimuli were isoluminant in the sense that the initial constriction, which is driven by rods and cones but not by ipRGCs, was identical. In other words, we selected brightness levels such that the red and blue stimuli mainly differed in their long-term effects on pupil size, while being comparable in most other ways. We consider this a relatively pure manipulation of pupil size. (Our constriction-based calibration procedure is only one of several possible procedures for matching brightness; for example, brightness can also be matched based on subjective brightness ratings or objective luminance as measured by a photometer. These different procedures likely all yield slightly different results. At present, we lack sufficient understanding of the underlying processes to say which, if any, of these procedures is optimal.) Crucially, and in line with Experiment 1, we found that the brightness of the tester was overestimated when the pupil was small, as compared to when the pupil was large. This effect was large and consistent between both sub-experiments (Experiments 2a and 2b).

To reiterate, we found that large pupils are associated with an underestimation of the brightness of the tester. (In our experiments, a brightness underestimation means that the tester stimulus had to be brighter than the referent stimulus for both stimuli to be perceived as equally bright.) This implies that perceived brightness not only influences pupil-size changes, but also that pupil size itself affects perceived brightness. Importantly, the direction of our effect is opposite from what you might expect and also from what was recently reported by Sulutvedt et al. (2021); that is, we found that large pupils are associated with an underestimation of brightness, despite the fact that they cause an increase in light influx.

We can consider our findings from the perspective of corollary-discharge theory (Sommer & Wurtz, 2008). This theory is especially important for explaining the difference between our own findings and those of Sulutvedt et al. (2021). According to corollary-discharge theory, when a movement is being prepared, a correlate of the motor command (a corollary discharge) is also sent to the visual cortex, thus allowing the visual system to take the perceptual consequences of the movement into account. In our study, we relied on pupil responses, which likely result in a corollary discharge being sent to the visual system. (Even though, to our knowledge, the existence of a corollary discharge for pupil responses has never been directly tested.) In turn, this may allow the visual system to “cancel out” the increased light influx that results from pupil dilation when making brightness estimations. In other words, a corollary discharge for pupil responses may serve brightness constancy, just like a corollary discharge for eye movements may serve visual stability across eye movements (Mathôt & Theeuwes, 2011; Wurtz, 2018). In contrast, in the study of Sulutvedt et al. (2021), pupil dilation was induced pharmacologically. This method likely paralyses the motor muscles of the eyes, thus bypassing the motor system altogether such that no corollary discharge is sent to the visual system. The lack of a corollary discharge might explain why, in their study, the increased light influx resulting from pupil dilation simply translated to an increase in perceived brightness.

The existence of a corollary discharge for pupil responses would explain why the world, under normal circumstances, does not seem to brighten when your pupils dilate. However, it does not readily explain why we found the opposite pattern: a “dimming” of the world associated with pupil dilation. One explanation for this may be that a corollary discharge is not perfect and leads to a slight overcompensation such that the visual system down-regulates its estimation of brightness more than it should after pupil dilation. At present, though, any interpretation is highly speculative, and we mostly intend it as a promising direction for future research.

There is no perfect way to experimentally manipulate pupil size while keeping everything else constant. This means that we cannot unambiguously attribute our findings to changes in pupil size. For example, in Experiment 1, brightness perception could also have been affected by error-related processing or other high-level cognitive processes. Similarly, in Experiment 2, it is possible that the blue and red stimuli affected brightness perception through other psychological processes, such as arousal. Therefore, future studies are recommended to use a variety of methods to manipulate pupil size that avoids involving high-level cognitive processes such as memory. If the results of multiple studies, even if each of them is imperfect by itself, point in the same direction, only then will we be able to draw firm conclusions about the effect of pupil size on subjective brightness perception. That being said, our own results as well those reported by Sulutvedt et al. (2021) are promising first steps that raise important questions, for example about the role of a corollary discharge in brightness constancy.

### Conclusion

Overall, the results from our experiments suggest that changes in pupil size can influence subjective brightness perception, at least in the absence of other cues from the immediate surroundings. When our pupils are small, we tend to overestimate the brightness of a stimulus. Conversely, when our pupils are large, we tend to underestimate the brightness of a stimulus. We observed this pattern when the pupils dilated in response to a red inducer (Experiment 2) and when the pupils dilated after an incorrect response (Experiment 1). We did not reliably observe this pattern when the pupils dilated due to increased memory load (Experiment 1); however, the effect of memory load on pupil size was also very small. We speculate that there may be a corollary discharge for pupil responses, which allows the visual system to take the perceptual consequences of pupil-size changes into account, albeit imperfectly, perhaps leading to overcompensation. Nevertheless, more studies that investigate the effect of pupil size on brightness perception are needed to confirm our findings. Studies in this area can lead to a new understanding of the relationship between pupil size and subjective brightness perception.

## Supplementary Material

Supplementary material
