# Supplementary material for: The influence of pupil responses on subjective brightness perception
Source: Perception. 2022 May 2;51(6):370–87. doi: 10.1177/03010066221094757 (PMC9121535; doi:10.1177/03010066221094757)
Supplement: Supplementary material [file sj-docx-1-pec-10.1177_03010066221094757.docx]

# Supplementary Material

## Pupil Size Differences by Memory Load and Performance Feedback

A supplementary Bayesian paired-samples t-test was conducted to compare the size of the differences in pupil-size changes between two conditions. First, the difference in pupil-size changes between low- and high-load conditions was calculated (Measure 1). Second, the difference in pupil-size changes after correct and incorrect feedback was calculated (Measure 2). Lastly, the difference values were compared with an alternative hypothesis Measure 1 < Measure 2. Data points above BF_10_ = 1 would suggest evidence supporting the alternative model. The resulting Bayes factor suggested extremely strong evidence in favour of the alternative model, BF = 30739.943. This result (Figure 12) indicated that Measure 1 (*M* = 0.1, *SD* = 0.089) was indeed smaller than Measure 2 (*M* = 0.228, *SD* = 0.138).

Figure 12. Differences of Pupil Size.


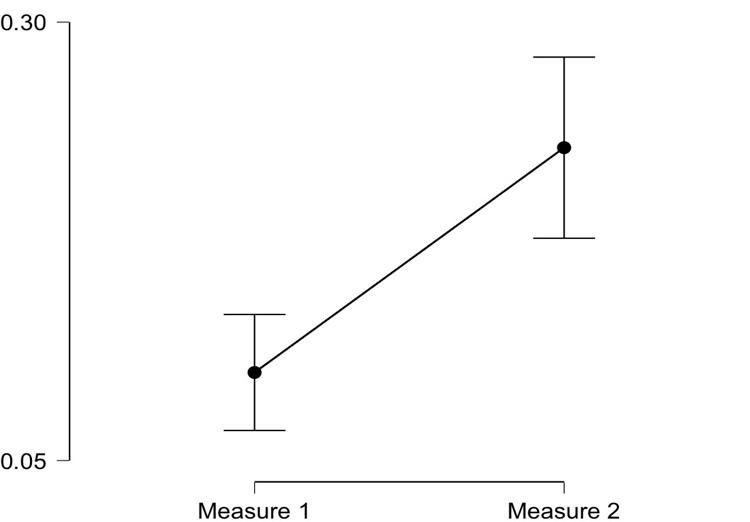


Note. The plot displays that pupil-size difference in Measure 1 (low-load vs. high-load) was smaller than that in Measure 2 (correct vs. incorrect). The error bars indicate 95% within-subject CI.
